# Impact of Total Parenteral Nutrition on Gut Microbiota in Pediatric Population Suffering Intestinal Disorders

**DOI:** 10.3390/nu14214691

**Published:** 2022-11-06

**Authors:** Tomás Cerdó, José Antonio García-Santos, Anna Rodríguez-Pöhnlein, María García-Ricobaraza, Ana Nieto-Ruíz, Mercedes G. Bermúdez, Cristina Campoy

**Affiliations:** 1 Maimonides Institute for Research in Biomedicine of Córdoba (IMIBIC), Reina Sofia University Hospital, University of Córdoba, 14004 Córdoba, Spain; 2 EURISTIKOS Excellence Centre for Paediatric Research, Biomedical Research Centre, University of Granada, 18016 Granada, Spain; 3 Department of Paediatrics, School of Medicine, University of Granada, Avda. Investigación 11, 18016 Granada, Spain; 4 Instituto de Investigación Biosanitaria Ibs-GRANADA, Health Sciences Technological Park, 18012 Granada, Spain; 5 Spanish Network of Biomedical Research in Epidemiology and Public Health (CIBERESP), Granada’s Node, Carlos III Health Institute, Avda. Monforte de Lemos 5, 28028 Madrid, Spain

**Keywords:** total parenteral nutrition (TPN), gut microbiota dysbiosis, pediatric population, inflammatory bowel disease (IBD), necrotizing enterocolitis (NEC), parenteral nutrition-associated liver disease (PNAD), TPN-associated mucosal atrophy, short bowel syndrome (SBS), intestinal failure (IF), postbiotics

## Abstract

Parenteral nutrition (PN) is a life-saving therapy providing nutritional support in patients with digestive tract complications, particularly in preterm neonates due to their gut immaturity during the first postnatal weeks. Despite this, PN can also result in several gastrointestinal complications that are the cause or consequence of gut mucosal atrophy and gut microbiota dysbiosis, which may further aggravate gastrointestinal disorders. Consequently, the use of PN presents many unique challenges, notably in terms of the potential role of the gut microbiota on the functional and clinical outcomes associated with the long-term use of PN. In this review, we synthesize the current evidence on the effects of PN on gut microbiome in infants and children suffering from diverse gastrointestinal diseases, including necrotizing enterocolitis (NEC), short bowel syndrome (SBS) and subsequent intestinal failure, liver disease and inflammatory bowel disease (IBD). Moreover, we discuss the potential use of pre-, pro- and/or synbiotics as promising therapeutic strategies to reduce the risk of severe gastrointestinal disorders and mortality. The findings discussed here highlight the need for more well-designed studies, and harmonize the methods and its interpretation, which are critical to better understand the role of the gut microbiota in PN-related diseases and the development of efficient and personalized approaches based on pro- and/or prebiotics.

## 1. Introduction

Parenteral nutrition (PN) is a very important nutritional support in infants and children when oral or enteral feeding routes are not possible or do not cover the high nutritional needs for a normal growth and development [1]. PN, as we know today, came into use for the first time in the 1960s, showing beneficial effects on the lean body mass preservation, growth and development, as well as the immune system’s development and function, while minimizing metabolic complications in patients with intestinal failure [2,3,4]. Although significant progress has been achieved over the last 50 years to make PN safe and effective, there are still some challenges associated with this form of nutritional support. Among them, doses of nutrients to be parenterally provided must be strictly implemented and monitored. This step acquires a vital importance to avoid the high risk of infections, metabolic disturbances or impair the liver function, which are associated with early or prolonged PN [5]. To counter this, several evidence-based guidelines about pediatric PN have been published and recently updated by the European Society of Paediatric Gastroenterology, Hepatology and Nutrition (ESPGHAN) and the European Society for Clinical Nutrition and Metabolism (ESPEN), supported by the European Society of Paediatric Research (ESPR) and the Chinese Society of Parenteral and Enteral Nutrition (CSPEN). Both guidelines provide clear patterns and evidence for its use in pediatric patients, including preterm and term neonates, infants and children [6,7].

Due to the considerable progress in the field of PN, this feeding route is widely used in infants and children for short- or long-term periods, at the hospital or at home, depending on different pathological situations [8,9,10]. PN is not only particularly important for preterm neonates who do not tolerate enteral feeds due to their gut immaturity and associated congenital or acquired gut disorders, including short bowel syndrome (SBS) {after massive intestinal resection due to necrotizing enterocolitis (NEC), intestinal atresia or gastroschisis}, but also for other patients affected by intestinal mucosal diseases (congenital diarrheal disorders), inflammatory bowel disease (IBD) or disorders of intestinal dysmotility (pediatric intestinal pseudo-obstruction) [11,12].

There is growing evidence that the aforementioned gastrointestinal disorders are directly or indirectly associated with microbial dysbiosis in the intestine [1], thereby seriously affecting its development and homeostasis maintenance [13]. The human gut microbiome, formed by approximately 1000 species, not only represents a major stimulus to the immune system, but also facilitates the performance of many physiological functions, especially during development [14]. Moreover, the gut microbiota is the most abundant type of antigen-presenting cells. Therefore, it is conceivable that total parenteral nutrition (TPN) may profoundly alter the gut microbiome composition and function, which could lead to detrimental effects on the intestine and significantly contribute to PN-associated liver disease (PNALD) development. In this sense, several studies have shown that the use of PN triggers changes in gut-associated lymphoid tissue functions, especially adaptative immune cells, which impair both the intestinal epithelium and chemical secretions. These events ultimately resulted in an intestinal microbiota dysbiosis and gastrointestinal (GI) barrier dysfunction against opportunistic pathogens [15]. TPN has been also associated with a significant loss of biodiversity and alterations in the pattern of the gut microbial colonization of infants over time, thus increasing the risk of adverse outcomes in the neonatal intensive care unit (NICU) [16]. This is particularly prevalent in preterm neonates since the critical stages of initial gut colonization occurs under several challenges (the high prevalence of a C-section delivery, a compromised health status, longer hospital stays in the NICU, the TPN and antibiotics therapy, among others) that can negatively affect their gut microbial colonization. In fact, preterm infants’ gut microbiota is characterized by a higher prevalence of *Proteobacteria*, a delayed establishment in its composition, as well as profound changes in the composition of intestinal short-chain fatty acids (SCFAs), which have been identified as high-risk factors for the development of neonatal infectious gastrointestinal diseases [17,18].

Taking into account previous considerations, this review highlights the current knowledge on the taxonomic and functional changes in the gut microbiota as a result of the use of PN in several gastrointestinal diseases in infants and children, including NEC, SBS and associated intestinal failure, IBD, parenteral nutrition associated liver disease (PNALD and cholestasis, among others) as well as TPN-associated mucosal atrophy. We also discuss the potential role of gut microbiota dysbiosis in the pathogenesis of these diseases that might serve as a noninvasive biomarker. By providing the perspectives of microbiota–host interactions in the aforementioned disorders, we offer an insight into the potential use of probiotics, prebiotics and synbiotics as promising effective prevention strategies and personalized treatments in those pediatric patients who have been receiving long-term PN.

## 2. Gut Microbiota Dysbiosis in Neonates Receiving PN

Over the last several decades, progress in perinatal and postnatal care have increased the survival of neonates, although morbidity later in life has increased [19]. Most investigative efforts have focused specifically on extreme preterm infants, given that around 50% of these neonates present neurologic and pulmonary complications, a three-fold increased risk of developing chronic kidney disease [20] and a low birth weight, as well as growth failure or postnatal growth restriction [21]. This raises the need for carefully designed early nutritional support, in terms of the optimal nutrient intake and the route of administration, to ensure a normal growth and a healthy development in preterm neonates, thus preventing early malnutrition-related adverse psychomotor and mental disorders later in life [22]. Optimal nutritional support is also mandatory in critically ill neonates who are admitted to the NICU due to these patients having limited macronutrient stores and relatively higher energy requirements [23]. In this sense, enteral nutrition (EN) is generally preferred for its additional physiological contribution in infant development and lower related complications [24,25]. Nevertheless, this type of nutritional support is not sufficient to cover the preterm infant´s needs due to their gastrointestinal tract immaturity and critically ill conditions. Consequently, TPN is often initiated to supplement the insufficient EN. It is well established that early- and long-term TPN in preterm and critically ill neonates show health benefits on the survival rates, an optimal weight gain in the NICU and the improvement of long-term neurodevelopment and motor development [26,27,28]; however, the specific health conditions of these patients make them more susceptible to neonatal morbidities, including bronchopulmonary dysplasia, late-onset sepsis, NEC-associated intestinal failure or the rapid onset of PNALD [1,28]. Thus, a balanced PN with an early and “aggressive” approach, either as a transition to or in combination with EN, must be used to limit the growth retardation and its related long-term consequences [29].

Recently, there is growing interest to evaluate the potential adverse effects of TPN on the gut microbiota’s composition and function, which is highly compromised in both critically ill and preterm infants who are usually exposed to aggressive treatments, the NICU environment or antibiotics, and how these changes may affect the development and progression of TPN-related comorbidities. Thus, for example, evidence suggest that preterm neonates with an increased risk of PNALD usually show a structural and functional gut microbiota dysbiosis and a subsequent potential “gut–brain axis” malfunction, immune system alterations and the development of non-communicable diseases during childhood and adult life [1]. In general, the studies mostly carried out in animal models support that PN dramatically changes the gut microbiota’s structure, with low abundances of *Firmicutes* and a high prevalence of *Bacteroidetes* and *Proteobacteria* phylum, as well as *Actinobacteria* phylum and *Akkermansia muciniphila*, but to a lesser extent [30,31]. Unfortunately, these changes adversely affect gastrointestinal health. On the one hand, it is well known that *Bacteroidetes* phylum promotes an intestinal inflammation and increases the intestinal permeability, which can drive a bacterial flux across the mucosa and result in a cytokines-mediated hepatocellular injury [32,33]. On the other hand, unlike *Firmicutes* phylum, *Proteobacteria* can metabolize the host-derived substrates in the absence of enteral feeding and incorporated them into gut microbial organisms, including *Enterobacteriaceae* of the *Proteobacteria* phylum, thus increasing its starvation resistance [34]. It is also important to highlight that PN increases the growth of opportunistic pathogens including *E. coli*, *Salmonella*, *Yersinia*, *Helicobacter* and *Vibrio* [35,36], and decreases the abundance of commensal microbials, such as *Bacteroides fragilis* [37]. All of these mentioned microbial compositional changes occur along with the PN-associated adverse effects on the gastrointestinal immunity as well as the cellular and chemical barriers, which in turn exacerbate intestinal failure and comorbidities during long-term PN [15].

In the light of these findings, there is no doubt that the understanding of the mechanisms and process derived from PN in critically ill neonates still raises many challenges and unique considerations. In fact, although the current guidelines support the safe use of this feeding route, improvements in the PN formulations, the timing of initiation, the advancement of nutritional support and clear individualized goals are still needed. For instance, a high risk of infection during TPN is considered one of the central current and future challenges in pediatric clinical research. In this regard, a higher risk of nosocomial infection noted among long-term TPN patients involves the need of prolonged antibiotic use, which has also been identified as a key factor in the gut microbiota’s modulation. Thus, recent data support that long-term antibiotic therapy profoundly decreased the relative abundance of potential probiotic candidates such as *Lactobacillus* and *Enterococcus* in those preterm patients receiving PN support [18]. These findings thereby support the need to take into account the duration of antibiotic therapy in the development of the optimal strategies for improving the gut microbiota’s composition. Consequently, the published guidelines by the Centers for Disease Control and Prevention [38] recommend extreme caution when performing a peripheral insertion to prevent intravascular catheter-related infections. Unfortunately, both the efficacy of these recommendations and the need for sterile barrier precautions during the subsequent changing of PN bags have yet to be thoroughly researched [39]. In this regard, specific strategies, such as the changing of PN bags every 48 h with the maximal sterile barrier precautions, seem to reduce the risk of bacteremia and mortality in preterm infants [40], but further randomized and controlled trials involving unmeasured or unknown confounders are still needed to verify its effectiveness. On the other hand, although its safety has been previously tested, another key challenge is to identify the most effective and useful probiotic strain in the prevention of severe NEC, late-onset sepsis and all-cause mortality in preterm neonates. This knowledge undoubtedly is of vital importance to better understand the exact mechanisms of action involved in the health beneficial effects of probiotics [41]. In fact, in a recent network meta-analysis of fifty-one randomized controlled trials (RCTs) involving 11,231 preterm infants, the overlap of strains with an effective result on multiple domains was not found, highlighting once again the need for more large and adequately powered RCTs aimed to evaluate the optimal probiotic-based treatment strategies [42].

## 3. Influence of Gastrointestinal Diseases in Infants and Children Receiving PN on Gut Microbiome: Potential Use of Pre-, Pro- or Postbiotics Therapies

### 3.1. Inflammatory Bowel Disease

The term inflammatory bowel disease (IBD) implies various chronic and relapsing inflammatory intestinal disorders with a low mortality, such as ulcerative colitis (UC), Crohn’s disease (CD) and IBD-unclassified (IBD-U), that primarily affect the small intestine and colon, although these disorders clearly differ in the location and severity of the lesion [43]. Both its incidence and prevalence are growing globally, and they are expected to continue increasing over the next few years, particularly in industrialized countries [44]. Previous epidemiological studies have also reported that about 25% of patients with IBD have their first symptoms in childhood and, subsequently, IBD incidence is greater in the pediatric population than the adult population [45,46]. As a result, IBD poses a major challenge for health care systems that are unable to deal with a staggering increase in the burden of this disease [47]. From an etiological point of view, IBD is defined as a multifactorial inflammatory or inflammation-associated disease that involves a complex interaction between genetic predisposition and immunological abnormalities, the gut microbiota and the environmental influences, although neither factor in itself is sufficient for IBD development [48,49]. Among these environmental factors, several perinatal (prenatal diseases, smoking during pregnancy and maternal age) and postnatal exposures (domestic hygiene, an urban environment, a diet high in proteins and total fats, infections and the abuse of antibiotics) have been clearly associated with IBD development [50]. However, the potential role of other perinatal factors such as prematurity and their potential relationship with various confounders in IBD development later in life remains unclear [51,52,53]. Recently, special interest has focused on the potential role of medical nutrition as a risk factor in patients with active IBD. The current ESPEN practical guidelines about clinical nutrition in IBD recommends EN based on formulas or liquids as a supportive therapy when oral feeding is not sufficient, while PN is only indicated in patients with advanced-stage and complicated disease [54]. This is of particular importance for preterm infants in which, due to their intolerance to enteral food, PN could be implanted shortly after birth to preserve the metabolic and hemodynamic stability. Nevertheless, studies with animals receiving PN showed a reduced gut growth, villous height, mucosal mass, protein mass, cell proliferation and mucosal immunity [55]. Furthermore, these deficiencies are implicated in the development of intestinal permeability, a bacterial translocation and a high risk of sepsis [56]. Despite the fact that these events may compromise the integrity of the gut in the neonate [57], the PN effects on IBD development remain unclear, and further well-designed clinical studies in humans are still needed [58].

It is well established that environmental, genetic and immune factors can directly or indirectly lead to gut microbiota dysbiosis, which has been proposed as the key risk factor for IBD development in pediatric patients and adults [59]. In this regard, the results obtained from large human cohort studies indicate that commensal bacteria from the *Firmicutes* and *Bacteriodetes* phyla as well a bacterial species from the genera *Bacteroides*, *Lactobacillus, Eubacterium*, *Faecalibacterium* and *Roseburia* are generally decreased in IBD patients. Conversely, these patients show a relative increase in the bacteria belonging to the phylum *Proteobacteria* (mainly *Escherichia coli*, *Enterobacteriaceae*, *Klebsiella* and *Proteus* spp.) and *Fusobacterium* [48,59,60]. In preterm infants, their poor somatic growth and subsequent need for PN support may further exacerbate the mentioned changes in the gut microbiota’s composition [61], but scarce information is available about this topic. Theoretically, the gut microbiota of PN-receiving patients should be characterized by the lower abundance of commensal bacteria (mainly *Bacteriodes* and *Bifidobacterium*) as well as the increased prevalence of potentially pathogenic Gram-negative bacteria [30,31,62]. These changes not only impair a healthy gut colonization, but also could interact with the host epigenome in order to predispose a gut infection and the high risk of diseases, including IBD [63,64]. Likewise, taking into account that the gut microbiota interacts with the host through metabolites, there is a growing interest to better understand the potential role of these signals in IBD development as well as their influence on immune maturation and homeostasis, the host energy metabolism and the maintenance of mucosal integrity [60]. In fact, patients with IBD presented alterations in their metabolite profiles as well as a perturbated interaction between the diet and gut microbiota. Moreover, specific classes of metabolites, particularly bile acids (BAs), lower levels of SCFAs (mainly acetate, butyrate and propionate) as well as the disruption of the tryptophan metabolism have been implicated in the pathogenesis of IBD [60,65,66]. Finally, it is important to note that these changes in the microbiota’s composition and gut metabolome involve a significant impairment in the host immune response, mucosal homeostasis and the energy metabolism, which further increases both the incidence and severity of disease in both adults and pediatric patients [59,60].

The above-mentioned results justify in themselves the therapeutic use of pro-, pre- and synbiotics in order to restore a healthy gut microbiota composition and ameliorate intestinal inflammation in IBD patients. This therapeutic option may be particularly crucial in preterm infants with an increased susceptibility to IBD and other gut dysbiosis-related diseases [67,68]. In fact, there is growing evidence supporting the use of different probiotics strains in the treatment of intestinal inflammation and IBD both in human and animal models [69], which is related to its modulatory effects on the growth of pathogenic bacteria, the immune response and the intestinal barrier activity. Thus, the studies which have been carried out have reported both the effectiveness and safety of treatments based on *Lactobacillus GG* [70], *Escherichia coli* Nissle 1977 [71], *Bifidobacteria* [72] and especially VSL#3 (a probiotic preparation of eight probiotic bacterial strains belonging to *Bifidobacterium, Lactobacillus* and *Streptococcus* genera) [73] in clinical remission in both active adult and pediatric IBD patients. Moreover, recent interest has focused on the potential use of commensal bacteria such as *F. prausnitzii*, *Akkermansia muciniphila* and *Bacteroides fragilis* in IBD treatment due to their ability to produce beneficial metabolites with anti-inflammatory effects in intestinal epithelial cells [74]. Conversely, the potential use of prebiotic as therapeutic agents in IBD is more limited. Nevertheless, promising clinical results, in terms of the restoration of normal gut microbiota, beneficial metabolites production and the modulation of the inflammatory response, have been obtained with treatments based on germinated barley foodstuffs, fructo-oligosaccharides and oligo-fructose-enriched inulin [75,76,77]. Interestingly, the current systematic review supports that the administration of synbiotics shows higher beneficial effects on gastrointestinal microbiota as well as remission, the disease activity index and the recurrence of IBD compared to those treatments based exclusively on pro- or prebiotics [78]. This assumption can also be supported by the fact that infant formulas supplemented with synbiotics may sustenance a more beneficial bacterial population closer to those reported in breastfed infants, which might promote long-term health benefits [79,80]. The use of paraprobiotics also appears of interest in the treatment of IBD, particularly in those patients with a compromised immunity. To date, in vitro studies suggest the potential therapeutic use of ultraviolet-inactivated LGG in this group of patients due to its ability to reduce the NF-kB-dependent expression of pro-inflammatory mediators [81,82,83]. In addition to these anti-inflammatory properties, some of the parabiotic proteins also seem to exert beneficial effects on the integrity of mucosa and intestinal walls, which are largely compromised in pediatric and adult patients suffering from IBD [84]. Nevertheless, there are no direct studies in IBD patients, and its clinical application remains to be investigated. Finally, there is growing evidence suggesting the use of postbiotics as a promising adjuvant treatment in patients with active IBD. Among them, the therapeutic use of SCFAs, mainly butyrate, acetate and propionate, has gained great interest due to strong association between the dysbiotic condition and impaired SCFAs-fermentative pathways in IBD patients. In this regard, a recent review carried out by Martyniak et al. [85] suggest that SCFAs supplementation in active phases of UC could have beneficial effects on both the patients’ well-being and clinical parameters (reduced pro-inflammatory and oxidative stress conditions associated with UC, lower intestinal bleeding and stool frequency). However, other studies have not found evidence for these therapeutic effects. On the other hand, tryptophan (Trp) and its metabolites (mainly indole acetate and propionate indole) have been also identified as a potential therapeutic agent in experimental colitis or IBD patients, which is supported by its immunomodulatory effects and the pivotal role in intestinal homeostasis via the activation of the aryl hydrocarbon receptor signaling pathway [85,86]. This potential therapeutic effect is also confirmed by profound changes in the Trp metabolism observed in animal models and patients with IBD [85]. Despite these findings, the effectiveness of a supplementation with Trp has been only reported in animal models of colitis, in which this type of treatment reduced the colitis-associated inflammatory condition and restored epithelial homeostasis, thus improving the recovery rate [87,88].

Overall, in light of these finding, it is important to note that, although a clear pattern of dysbiosis has been associated with the development of IBD, there is still controversy regarding whether this is a causal effect or a consequence of the pathology [59]. Likewise, the IBD-associated dysbiosis pattern may vary among patients due in part to the variation in the sample type, sample location and the disease status of the subjects, as well as the materials and methods of the analysis. Finally, the potential relationships between the PN and gut microbiota dysbiosis in IBD patients also need to be evaluated. Consequently, large randomized controlled trials in pediatric patients, especially in preterm infants, and well-designed animal models are still needed to better understand the role of dysbiosis in IBD and its mechanisms of action, which will allow us to design new gut microbiome-based therapeutic strategies for the treatment of this disease.

### 3.2. Necrotizing Enterocolitis (NEC)

Necrotizing enterocolitis (NEC) is one of the most frequent and fatal intestinal disorders in preterm infants which is characterized by variable intestinal injury, from epithelial injury to transmural involvement and perforation, accompanied by intestinal inflammation and often bacterial invasion [89]. While its incidence is extremely rare in term infants, and usually related to congenital anomalies, sepsis or hypotension, NEC affects about 5–12% of neonates born at a very-low birth weight (VLBW; <1500 g) [90], with an associated mortality rate of nearly 30–50% for preterm infants of an extremely low weight (<1000 g) and 10–30% for VLBW neonates [91]. Its pathogenesis has been charged to a multifactorial origin marked by a low gestational age and weight at birth, formula feeding and intestinal dysbiosis, although maternal factors such as chorioamnionitis, a high BMI, preeclampsia, or smoking during pregnancy also seem to be involved in NEC development [92,93,94].

Recently, there is growing evidence supporting the significant relationship between NEC onset and progression and gut microbiota dysbiosis. In this regard, gut microbiota from preterm neonates suffering from NEC is characterized by a low bacteria diversity and commensal bacteria abundance, as well as an overgrowth of pathogenic bacteria causing concomitant infections [95]. However, due to heterogenicity in molecular methods used for identification and detection, no common microbial pattern has been consistently identified. While Mai et al. [96] reported a high abundance of *γ-Proteobacteria*, Normann et al. [97] found an increased abundance of *Bacillales* and *Enterobacteriaceae* in the stool samples obtained from preterm infants who suffer from NEC. Moreover, patterns of gut microbiota dysbiosis seem to change according to the time of the NEC onset, with a high *Firmicutes* and *Clostridia* abundance reported in its early onset in contrast to the predominance of *Entorobacteriaceae*, *Escherichia/Shigella* and *Cronobacter* in those cases of later onset NEC [98,99]. It is also important to note that NEC-related gut microbiota dysbiosis may occur several weeks prior to the onset of the disease, suggesting a time frame in which gut microbiota-targeted therapy could positively influence the clinical outcomes [100].

In addition to these changes in the gut microbiota’s composition, the studies carried out to date suggest a potential involvement of specific genetic variants regulators in NEC pathogenesis, including the nuclear factor κB1 (NF- κB1), the co-receptor molecule lymphocyte antigen 96 (MD-2 co-receptor), the small glycolipid transport protein ganglioside GM2 activator and the interleukin (IL)-1 related receptor (IL-1R). These genetic variations are related to the upregulation of the Toll-like receptor-4 (TLR-4)-dependent signaling pathway, thus increasing the intestinal inflammatory response [101,102]. Interestingly, TLR4 activation also leads to the impairment of the epithelial barrier and a subsequent luminal bacterial translocation, which results in the recognition of gut the microbiota by TLR-4 expressed in mesenteric blood vessels, favoring vasoconstriction, intestinal ischemia and NEC [103]. All of these changes, along with the presence of unusual intestinal microbial species and the overall reduction in the gut microbiota’s community diversity, may explain why preterm neonates who develop NEC also have a high susceptibility to infectious diseases [104].

As mentioned above, both the mode of feeding (enteral versus parenteral feeding) received by preterm infants and how the transition between both of the feeding routes occurs, also seems to be involved in pathogenesis and the clinical outcomes of NEC. Thus, studies performed in animal models suggest that long-term TPN may predispose to TLR-4-dependent NEC lesions [105]. Likewise, clinical strategies based on acute enteral refeeding seem not to be effective in preventing small intestinal mucosa homeostasis, including intestinal epithelial cell apoptosis, the loss of the epithelial barrier function and the failure of the leucine rich repeat-containing G protein-coupled receptor 5-positive stem cell expression [106]. Overall, these findings also provide evidence showing that that a switch from parenteral to enteral nutrition may rapidly induce diet-dependent histopathological, functional and proinflammatory insults to the immature intestine. Consequently, special attention should be given to the speed of feeding progression, although the results achieved to date are contradictory. In fact, Roze et al. [107] observed that a higher speed of feeding progression is not a risk factor for NEC but relates rather to a shorter time with PN. Conversely, Ou et al. [108] found no effects of faster advancing feeds on late-onset incidence. Moreover, although PN is initiated in *nil per os* patients (or “nothing to mouth”) following the NEC diagnosis [108], it is also important to highlight that this feeding route at the NECs onset seems not to improve the clinical outcomes (the rates of surgical intervention or in-hospital mortality) in those who are premature with a low birth weight [109].

To date, due to its multifactorial pathogenesis, there is no optimal treatment for NEC, and therefore reducing its high incidence, morbidity and mortality in prematurely born neonates still remains a major challenge in pediatric research. The classical therapeutic approaches are largely aimed at preventing gut microbiota dysbiosis through well-designed feeding protocols in which breast milk is preferred and moderate antibiotics are used, as well as the routine administration of pro-, prebiotic or both agents [110,111]. Nevertheless, new therapeutic strategies which involve immunological approaches have been also suggested, but its efficacy and safety in well-designed clinical trials are still under study [112]. Among all of these mentioned treatments, a supplementation with pro- or prebiotics has emerged as a promising strategy to reduce NEC incidence in preterm infants receiving parenteral nutrition [113,114,115]. In this regard, a recent meta-analysis that included data of more than 50 RCTs involving 10,812 very preterm or very low birth weight (LBW) infants suggests that probiotic supplementation (mainly based on *Bifidobacterium* spp., *Lactobacillus* spp., *Saccharomyces* spp. and *Streptococcus* spp., either alone or in combination) seems to reduce the risk of NEC, mortality and a late-onset invasive infection [116]. Interestingly, Nandhini et al. [117] reported that breastmilk in combination with enteral synbiotic supplementation based on *Lactobacillus*, *Bifidobacterium* and fructo-oligosaccharides (FOS) significantly reduces the incidence of NEC at all stages in preterm infants. However, any effects on the NECs severity, NEC-associated sepsis or mortality were not found. Despite these beneficial effects, long-term probiotic-based therapy may have negative effects both in preterm and critically ill infants due to their immature intestinal barriers, impaired immune function or their high risk of sepsis [85,118]. Hence, new therapeutic strategies focused on preventing gut microbiota dysbiosis in patients suffering from NEC are still needed. In this regard, the therapeutic use of postbiotics or paraprobiotics has recently emerged as a promising strategy. According to the International Scientific Association of Probiotics and Prebiotics (ISAPP), postbiotics are defined as ‘preparation of inanimate microorganisms and/or their components that confers a health benefit on the host’ [119]. Among them, butyrate may have a potential use in the treatment and prevention of NEC due to its beneficial effects on intestinal growth and the differentiation, inflammatory suppression and regulation of apoptosis [118]. In addition to these direct effects, studies also suggest that the use of butyrate in combination with pro- or prebiotics enhances the beneficial effects on gut microbiota, thus supporting butyrate-based therapy as a safe and potentially highly effective option in the treatment of NEC and other gastrointestinal diseases [118]. On the other hand, the term paraprobiotic refers to the ‘non-viable microbial cells or raw cellular extracts with beneficial health effects when they are administered in adequate amounts’ [120]. For example, a study carried out in murine models of immature intestines reported that the use of heat-killed *Lactobacillus rhamnosus* GG (LGG) not only improved the intestinal barrier maturation, but also showed a significantly less mortality and risk for adverse outcomes compared to live LGG, suggesting its potential use as a promising alternative to live probiotics [121]. However, the results obtained to date do not provide evidence of the sufficient quality and applicability to use probiotics, prebiotics, postbiotics or paraprobiotics in clinical practice, which determines the need for further large, well-designed RCTs. Moreover, in order to evaluate the correct PN use during NEC development, these studies should be designed to prevent an uncontrolled confounding bias. To make the right choice of time of sample collection in relation to the NEC onset is also of crucial importance to obtain robust conclusions about the temporal relation between the colonization by a specific bacterial strain and the NECs onset. Lastly, as mentioned above, each neonatal intensive care unit must standardize the feeding protocols and ensure that they are consistently followed.

### 3.3. Parenteral Nutrition-Associated Liver Disease (PNALD)

It is well established that long-term PN (>27 days) causes a complex wide spectrum of liver function alterations, commonly named PNALD, which is also referred to as PN-associated cholestasis (PNAC), PN-associated liver injury (PNALI) or, more recently, intestinal failure-associated liver disease (IFALD). Among these concepts, both PNALD and PNAC usually refer to liver disease related to the potential toxic compounds present in PN, while IFALD is normally used to specify the hepatobiliary dysfunction caused by intestinal failure [122].

PNALD/IFALD are clinically manifest with intrahepatic cholestasis (conjugated bilirubin levels > 2 mg/dL) in the absence of any other liver etiology, hepato-steatosis and altered biochemical markers of liver damage. These hepatic complications can lead to fibrosis and cirrhosis in those cases with prolonged PN, which can variably progress to end-stage liver disease and thus requiring a liver transplantation, or death [123,124]. Its reported incidence varies considerably depending on the diagnostic criteria used and the age groups, with a higher incidence in infants and child patients (25–60%) compared to adults (15–40%). Moreover, its incidence is particularly common in LBW premature infants with long-term PN (>85%) [125,126]. Consequently, research efforts have primarily focused on elucidating the complex pathophysiological mechanisms involved in PNALD/IFALD and its most effective treatment. In this sense, PNALD/IFALD has a multifactorial origin involving both nutrition-, patient- and nutrition-related risk factors [1,125]. Among the latter, the immaturity of the liver function in preterm and LBW neonates, there is a high risk of recurrent bacterial infections and NEC observed on these patients, as well as SBS and the associated intestinal comorbidities, have been identified as potent risk factors for PNALD/IFALD development [124,127,128,129].

Regarding the nutrition-related risk factors, the inability to successfully implement enteral nutrition in preterm and critically ill infants, and consequent long-term PN, have been established as key factors for PNALD/IFALD development [123,127,129]. In fact, the lack of EN impairs a gastrointestinal hormones secretion (gastrin, motilin, secretin and glucagon, among others), thus leading to important abnormalities in intestinal motility, gallbladder contractility, enterohepatic circulation and bile acids secretion/absorption, all of which potentially increase the risk of cholestasis and subsequent PNALD/IFALD [124,127,130]. On the other hand, prolonged PN also adversely impacts the hepatobiliary system as a direct consequence of the immaturity of organ systems and their resulting inability to detoxify certain toxic minerals (mainly aluminum, copper and manganese) present in parenteral products, causing or aggravating cholestasis [123,129]. However, other components and nutritional features of PN have been also implicated in mediating PNALD/IFALD pathogenesis, including an excessive calorie intake, a high protein content (>2.5 g/kg/day) as well as certain amino acid deficiencies (taurine, choline and glutamine) in PN solutions [123,129,131]. Nevertheless, both sources and amounts of intravenous lipid emulsions (ILEs) have acquired the greatest interest as key factors in PNALD/IFALD pathogenesis [124,127]. Compared to fish oil-based ILEs (FO-ILEs), traditional soybean oil-based ILEs (SO-ILEs) are strongly discouraged due to the hepatotoxic effects caused by its high abundance in phytosterols, plant-based cholesterol-like compounds and pro-inflammatory omega-6 polyunsaturated fatty acids (ω-6 PUFAs). Moreover, SO-ILEs also contain relatively low amounts of the antioxidant α-tocopherol as well as anti-inflammatory ω-3 PUFAs (mainly docosahexaenoic (DHA) and eicosapentaenoic (EPA) acids) [125,126,129]. Due to these nutritional characteristics, long-term PN programs using SO-ILEs have been associated with altered bile acid homeostasis, reduced cholesterol synthesis and bile flow. All of these conditions ultimately promote hepatic and liver inflammation via macrophage-derived IL-1β/NF-kB signaling, as well as cholestasis, in both pediatric patients [132,133] and murine models of human IFALD [134,135].

Based on the pathophysiological mechanisms described above, preventive and therapeutic strategies must be aimed to avoid long-term PN programs and parenteral lipids, re-establishing oral feeding when possible. However, the alternative therapeutic approaches (cyclic PN, ursodeoxycholic acid-based therapy, lipid restriction and/or replacement, surgical procedures or organ transplantation in severe cases of IFALD), should be considered in those cases where PN is really needed [123,124,125,126,127,129,136]. Among these approaches, both lipid restriction and replacement-based strategies have been successfully evaluated for the treatment or prevention of PNALD/IFALD, which is consistent with the aforementioned role of SO-ILEs in disease pathogenesis. In this regard, although SO-ILEs treatment has been traditionally based on the dose of 2–3 g/(kg·day), the studies carried out to date have shown that the use of reduced lipid doses [<1 g/(kg·day)] is strongly related to lower PNALD incidence [137]. These promising results should be considered with caution due to the fact that the beneficial effects have not been observed in preterm infants with a low risk of PNALD/IFALD [16,138]. Moreover, treatment based on reduced SO-ILEs doses may provide an inadequate supply of DHA and arachidonic acid (AA)s, further increasing the risk of fatty acid deficiencies in preterm infants with subsequent potential negative long-term effects on the brain’s growth and neurocognitive development. Nevertheless, these potential deleterious effects associated with lipid restriction-based therapy are still unknown and more studies are required [125]. The current guidelines prepared by the ESPGHAN/ESPEN/ESPR/CSPEN working group on Pediatric Parenteral Nutrition not only recommend both the discontinuation of or the reduction in SO-ILEs dosage, but also encourage the use of mixed lipid emulsions largely based on fish oil (FO) in the treatment and management of PNALD/IFALD in preterm infants. These recommendations are supported by the beneficial effects of FO on hepatosteatosis and its lower accumulation in the liver, as well as its nutritional composition (rich in ω-3 PUFAs and tocopherols and lack of phytosterols). Furthermore, short-term FO monotherapy programs should be only used as a rescue treatment in those cases with severe IFALD [137].

Despite the several proposed mechanisms and therapeutic strategies, the high incidence and fatal complications of PNALD/IFALD in preterm infants and in critically ill patients support the need to evaluate the novel factors involved in the onset and progression of disease, facilitating the development of more effective treatments. Thus, growing evidence suggests the important role of “gut–brain axis” in PNALD/IFALD pathogenesis [1], which is strongly related to bidirectional communication between both organs. On the one hand, liver products such as bile salts, antimicrobial molecules and metabolites are transported to intestinal lumen via the biliary tract and/or capillary system, where they exert their physiological and modulatory functions on gut microbiota, the intestinal barrier’s integrity, hepatic bile acids synthesis, energy utilization and both glucose and lipid metabolisms. On the other hand, metabolites produced by host and gut microbial communities circulate through the portal vein to the liver and, consequently, can also modulate its functions [128,139,140]. As result, long-term PN may not only cause a direct liver injury but it can also affect gut homeostasis, disrupting the enterohepatic axis and causing indirect liver damage that promotes PNALD/IFALD progression. In this sense, studies performed on both animal models of PNALD and human patients (preterm infants and adults) support that PN induces dramatic changes in the gut microbiota’s composition, but these changes vary between the mentioned groups. Overall, PN-associated gut microbiota dysbiosis is characterized by a low bacterial diversity and the high abundance of potential pathogenic bacteria such as *Proteobacteria* and *Bacteriodetes* at the expense of beneficial commensal bacteria, mainly *Firmicutes* [90]. However, gut dysbiosis may be more relevant in the preterm gut due to its higher sensitivity to postnatal life events and, therefore, reduced gut adaptation. Thus, a relative *Proteobacteria* abundance may represent nearly 70% of gut microbiota communities in those preterm infants who developed PN-associated cholestasis [16]. Interestingly, a prospective two-center study performed by Parm et al. [24] reported that gut a colonization pattern in preterm infants with PN is largely based on reduced Gram-positive and Gram-negative bacteria, the high abundance of *Candida albicans* and the low mucosal colonization by *Enterococcus faecalis*, an intestinal lactic acid (LA) bacterium with potent immunomodulatory effects on Toll-like receptors (TLR)-dependent signaling pathways. In view of these findings, Cahova et al. [1] proposed a general mechanism to better understand the potential role of the gut microbiota-related factors on PNALD/IFALD pathogenesis. In addition to the direct effects of PN on the gut microbiota, these authors suggest that the aforementioned shift to pathogenic bacteria may be encouraged by PN-dependent changes, such as: (i) an altered gut barrier function (decreased mucin secretion and tight junctions’ protein expression); (ii) an decreased IgA response; and (iii) the impaired antimicrobial function of Paneth cells. Irrespective of the cause, the high abundance of potential pathogenic bacteria triggers the TLR-dependent signaling pathway and the subsequent pro-inflammatory cytokines secretion, further impairing the epithelial barrier function. As a consequence, both pathogenic bacteria and its endotoxins may reach the liver through portal circulation, thereby leading to the suppression of bile acid transporters and, ultimately, hepatic and liver damage [1,128]. Both human and animal models of PNALD also suggest that PN-associated gut dysbiosis is also characterized by the low abundance of short-chain fatty acids (SCFAs)-producing bacteria, such as *Ruminococcaceae* and *Lachnospiriaceae* [141,142]. The resulting low SCFAs production may negatively impact the immune response in terms of decreased B-cell maturation and a specific antibody production, further increasing the susceptibility to pathogenic bacteria and subsequent damage in the epithelial barrier’s function. As a final consequence, these changes promote bacterial translocation and liver damage [128]. Lastly, and despite the few studies conducted so far, the type of lipid emulsions used may also affect the gut microbiota’s composition in pediatric patients receiving PN. In this regard, using animal models of TPN-induced intestinal mucosa atrophy, Feng et al. [143] suggested, for the first time, the potential interaction between the ILEs used in PN and gut microbiota. These authors showed the beneficial effects of olive oil-based emulsion on the gut epithelial’s integrity and inflammatory state, compared to SO-ILEs or a combination oil-blend emulsion (with 15% of FO). Likewise, Harris et al. [144] reported that SO-ILEs induced shifts within the gut microbiota in terms of the high prevalence of the bacterial family *Erysipelotrichaceae*, a Gram-positive bacterium, which directly or indirectly stimulates the TLR4 pathways, and a subsequent liver injury. Recently, research efforts have focused on the role of the ω-3: ω-6 PUFAs ratio in lipid emulsion on the gut microbiota’s composition. Thus, predominantly ω-3 PUFAs-rich ILE is associated with a significant increase in the relative abundance of bile-acid tolerant Gram-negative *Enterobacteriaceae*, while ω-6 PUFAs-rich PN showed a specific and significant increase in *Parabacteroides* [145,146]. Interestingly, the use of FO-ILEs has the ability to modify the gut microbiota in different intestinal segments, with the high abundance of *Bacteroidaceae* in ileum, and *Rikenellaceae* and *Ruminococcaceae* in the colon, thus alleviating intestinal liver damage [147]. However, although this knowledge supports the aforementioned role of lipid restriction and/or replacement in the treatment and prevention of PNALD/IFALD, the mentioned studies did not assess and present their microbiome analyses in detail. Moreover, the results were largely obtained in animal models, and further well-designed RCTs in both pediatric and adult patients are still needed to discuss its implications in a clinical context.

Although the mechanistic role of gut microbiota and the modified intestinal environment in the PNALD/IFALD progression remains unclear, the proposed model opens the possibility of using pre-, pro- and/or synbiotics as promising therapeutic strategies for the PNALD/IFALD treatment. To date, probiotic bacteria belonging to the genera *Lactobacillus* and *Bifidobacterium* are widely used in the treatment of diverse gastrointestinal diseases, including antibiotic-associated diarrhea, NEC and inflammatory bowel disease [148,149,150,151]. These therapeutic effects are not only due to their ability to produce lactic and acetic acids, but also their modulatory effects on SCFAs-producing commensal bacteria, thus inhibiting the growth of pathogenic bacteria [152]. Nevertheless, the effectiveness of a pro-, pre- and/or synbiotic-based treatment has remained controversial in patients with PNALD/IFALD or a high risk of disease. In this regard, Sentongo et al. [153] carried out a double-blind, placebo-controlled randomized crossover clinical trial which aimed to evaluate the therapeutic effects of *Lactobacillus rhamnosus* on the intestinal permeability in children with SBS and at a high risk of PNALD/IFALD; however, no beneficial or detrimental effects were found after a probiotic treatment for 4 weeks. Interestingly, synbiotics therapy based on *Bifidobacterium breve*, *Lactobacillus casei* and galacto-oligosaccharide seems to have beneficial effects in pediatric SBS treatment by increasing fecal SCFAs as well as normalizing the height and weight velocity [154]. Finally, a systematic review performed by Reddy et al. [155] suggested the potential adverse effects which are related to probiotic therapy in terms of *Lactobacillus* sepsis and D-lactic acidosis. Furthermore, therapeutic strategies based on other probiotics strains, including lactate-producing bacteria and strictly anaerobic butyrate producers (mainly *Lachnospiraceae* and *Ruminococcaceae*), are strongly discouraged due to their potential negative effects on the gut microbiota’s composition and short bowel environment, respectively. Overall, a better knowledge about the role of “gut–liver axis” in PNALD/IFALD pathogenesis, as well as changes in the gut microbiota’s composition associated with disease severities and PN duration, is a critical step to select efficient and personalized approaches based on pro- and/or prebiotics for PNALD/IFALD treatment.

### 3.4. Gut Mucosal Atrophy

It is well known that gut mucosal has the ability to respond to a wide range of internal and external environmental stimuli through diverse physiological, cellular and molecular mechanisms controlling its morphology and function. Therefore, mucosal adaption is critical to gut homeostasis and the subsequent host health [156]. However, specific pathological or nutritional conditions (the absence of enteral nutrition as well as long-term periods of starvation or parenteral nutrition) induce gut mucosal atrophy. This condition is mainly characterized by a marked decrease in the intestinal function and profound morphological changes in terms of a decreased villous height, crypt depth, surface area and epithelial cell numbers [157]. In this regard, the results obtained from animal models support an association between TPN and mucosal atrophy, even if this route of feeding is properly provided as a life-support system for neonates, infants and children with gastrointestinal disorders [158]. Overall, TPN-associated mucosal atrophy is mainly caused by nutrients deprivation in the luminal content and subsequent mucosal hypoplasia via the TNF-α/EGF signaling pathway; nevertheless, intestinal barrier dysfunction is also involved through different mechanisms such as an altered peristaltic compression and villus motility, a decreased enterocytic proliferation/differentiation and an increased enterocyte apoptosis. Taken together, these changes result in a loss of the overall barrier function and subsequent bacterial translocation [158]. Moreover, there is also growing evidence showing that gut mucosal atrophy is driven by TPN-related gut microbiota dysbiosis, characterized by a decreased α-diversity, a lower abundance of *Firmicutes* as well as an increased prevalence of potentially pathogenic Gram-negative bacteria, mainly belonging to *Proteobacteria* phylum [1,16,159,160,161]. Interestingly, the studies carried out to date also suggest that these changes in bacterial diversity and richness are positively related to the longer duration of parenteral nutrition and its related consequences, such as the lack of fermentable fiber and the depletion of beneficial SCFAs, further increasing the abundance of potentially harmful bacteria [16,159,161,162]. This TPN-related shift in the gut microbiota’s composition is strongly suspected to trigger a TLR-dependent proinflammatory response in the gut with the consequence being a loss in the epithelial integrity, thus causing morphological alterations, and a loss in the barrier function [1].

Significant efforts have been made to improve or prevent TPN-associated intestinal mucosal atrophy. In this sense, the studies performed in animal models of gut mucosal atrophy reported that the use of different growth and stimulation factors, including the epithelial growth factor [162], glucagon-like-protein-2 [163], hepatocyte growth factor [164], ghrelin [165], glutamate [166], arginine [167] or PUFA emulsions [168,169], can improve intestinal development and its function, thus preventing TPN-associated intestinal mucosal atrophy. Interestingly, prebiotic-, probiotic- and postbiotic-based treatments have been recently used in order to achieve this purpose. Thus, the experimental studies conducted in both rodents and piglets that received PN supplemented with SCFAs, mainly butyrate, reported lower rates of infection associated with an improved mucosal immunity [170]. Likewise, the therapeutic use of PN enriched with butyric acid is also supported by its moderate but positive effects on the recovery of intestinal mucosa [171,172] and mucosal protein synthesis [173]. Moreover, using a piglet model of intestinal failure, treatment based on partial EN supplemented with short-chain fructo-oligosaccharides (scFOS), was more effective than a probiotic treatment with *Lactobacillus rhamnosus* GG for an intestinal adaptation [174]. Although these results are promising, studies on TPN-associated gut mucosal atrophy and the potential use of pre- and probiotic therapy in humans are very limited, particularly in children and even more in premature ones. This may be due to the fact that those children who require TPN also have a high incidence of gastrointestinal disorders. Therefore, it is certainly difficult to discriminate whether gut mucosal atrophy is the cause or consequence of the type of nutrition received or the existing gastrointestinal disease. Consequently, further studies are needed to clarify the potential relationship between TPN, gut mucosal atrophy and gut microbiota dysbiosis in humans, which will open up new clinical and therapeutic avenues based on the use of pre- and probiotics.

### 3.5. Short Bowel Syndrome (SBS) and Intestinal Failure (IF)

Together with congenital diseases of enterocyte development and severe motility disorders, it is well established that all the above-mentioned pathologies can ultimately lead to intestinal failure (IF). Clinically, IF is defined, both in adult and pediatric patients, as the reduction in the gut’s function or mass below a minimum needed to absorb nutrients and fluids, such that an intravenous supplementation with PN is required to maintain health and/or growth [175,176]. Thus, in clinical practice, the degree of IF may be indirectly measured by the level of PN required for a normal or catch-up growth [177], where chronic IF is defined by the need for PN for >60 days due to an intestinal disease, dysfunction or resection (SBS) [160,178]. However, in pediatric population, SBS has been identified as the leading cause of IF, with an estimated incidence of 24.5 cases per 100,000 live births, and its incidence is markedly greater in premature infants [178,179]. From a clinical point of view, pediatric SBS frequently occurs after either a surgical resection of specific anatomic or physiological abnormalities of the small intestine or to impairments of the intestinal function, including pseudo-obstruction or villous atrophy [180,181]. Consequently, the intestine is either too short or dysfunctional, despite it being of an adequate length. Overall, pediatric SBS is strongly associated with NEC, midgut volvulus, gastroschisis, intestinal atresia and extensive Hirschsprung disease [178,182], and it is usually following an extensive surgical resection, leaving the small bowel (SB) length below the critical value for an adequate nutritional supply. A severely reduced mucosal surface results in malabsorption with subsequent diarrhea, a water–electrolytes imbalance and malnutrition [175,183]. At birth, term-neonates have an SB length of approximately 250 cm and their intestines lengthen substantially during their first year of life [184]. Preterm infants have a greater potential for bowel growth since their intestines lengthen substantially during the last trimester of gestation [185]. The cut-off length for SBS is related to several factors. In general, SBS occurs after a massive resection, leaving less than 40 cm of viable SB. A residual bowel length of only 15–40 cm has been associated with bowel adaptation, intestinal autonomy and PN weaning, but there is a lack of information regarding the long-term growth [186,187].

Regarding the prognosis, several factors can determine SBS, including an underlying diagnosis, the type of segments preserved, the presence of the the ileo-caecal valve (ICV) and the colon, a long-term stoma vs. a primary anastomosis, the number of surgical procedures, as well as the patient’s age at the time of surgery. Other factors, such as the functionality of the residual bowel, especially in motility disorders, are also relevant to SBS development [178]. In many cases, SBS is fatal as a result of associated IF and, although effective treatment based on intestinal rehabilitation programs is possible [179], these children may suffer from serious complications, such as catheter-related bloodstream infections, the loss of venous access, small intestinal bacterial overgrowth, and IFALD [178,188]. While IFALD has been identified as a negative predictor for survival [127,189], other factors such as gestational age, diagnosis, residual small- and colon bowel length and remaining ICV have been recognized as positive predictors for enteral autonomy in pediatric IF [190,191,192]. It is also important to note that IF may be reversible or irreversible, depending on the underlying cause of SBS, the length of the remaining intestine or the treatment used to develop or restore the intestinal capacity. Although severe and even irreversible IF in children is very challenging, patients with SBS may undergo intestinal adaptation, where the remaining small intestine undergoes structural and functional changes to increase its absorptive capacity [193]. In this regard, small-bowel resection (SBR) is associated with a rapid adaptation and improved clinical outcomes. However, jejunocolic anastomosis and jejunostomy are the most common surgeries resulting in clinical IF, usually because of NEC disease-related or distal small bowel lesions. One major gap in clinical management is the strategies used to avoid an attenuated functional ability of the remaining bowel with management decisions that can be controlled: diet, probiotics, anti-secretion medications and/or oral antibiotics. After the intestinal resection, an early expansion of the secretory cell lineages, including the Goblet and Paneth cells, occurs while the number of absorptive enterocytes increases at a later time point. Early hemodynamic alterations also may contribute to local angiogenesis as well as an increased tissue oxygen utilization. These changes support mucosal growth, leading to an increase in transporter cells, and promoting a slower bowel transit time, ultimately enhancing the absorptive capacity of the remaining bowel. Multiple factors can enhance the intestinal adaptation of the small bowel, including anatomic features, intraluminal nutrients, gastrointestinal (GI) secretions and the systemic factors [194], eventually allowing patients to wean off PN and become fully dependent on enteral and oral feeding [177]. Due to technical refinements and steady advances in the development of highly sophisticated nutrient solutions consisting of optimal combinations of macronutrients and micronutrients, PN plays an important role in patient management [195]. Indeed, PN has become a safe and efficient feeding technique [178].

Regardless of its cause, recent studies have also reported an altered gut microbiota composition in patients with IF, which is characterized by a decrease in bacterial diversity [196,197,198], as well as an increase in the abundance of pathogens diversity [196,197,198,199,200,201]. Moreover, it seems likely that the absence of luminal substrate which is essential for bacterial growth and sequential “gut starvation” alters the production of SCFAs in patients receiving PN, thus affecting the gut’s vascular flow and motility, cell proliferation and differentiation [198,202]. Consequently, gut microbiota dysbiosis has been associated with adverse clinical outcomes in IF, including bacterial translocation, D-lactic acidosis, a central line-associated bloodstream infection, poor growth and liver disease [197,198,200]. Interestingly, the gastrointestinal tract (GI) is divided into different functional sections with specific environmental conditions, leading to a different composition of the gut microbiota depending on the GI location. For instance, *Lactobacillus, Veillonela* and *Helicobacter* are predominant in the proximal gut; on the other hand, *Bacilli, Streptococcaceae* and *Actinobacteria* are increased in the duodenum, jejunum or ileum, respectively, while a high population of *Lachnospiraceae* and *Bacteriodetes* are found in the colon [203,204]. Consequently, a specific pattern of gut microbiota dysbiosis can be detected depending on the location and extent of SBS. Nevertheless, to date, the studies carried out have mainly focused on fecal bacteria’s composition, but information about specific changes in the bacteria population according to the affected GI section is unknown. It is important to highlight that intestinal resection also reduces the diversity of the microbiota which is present in the remnant bowel and the colon [142,205]. The surgical procedure itself induces changes in the microbiome, likely resulting from exposing the bowel lumen to oxygen and temporarily interrupting the local blood flow. Depending on the length and location of the bowel resected, the loss of the intestine also may induce long-term changes, such as a lower fecal pH, a faster transit time and/or altered pancreatic-biliary secretions. These changes modify the gut environment and can trigger the prevalence of certain Gram-positive bacterial communities, such as the facultative anaerobe *Lactobacillus* [206]. The impact of surgery on the host–microbiota balance acts at multiple levels, influenced by physiological stress of surgery, fasting and the antibiotic treatment [207]. Dysbiosis also corresponds with a decrease in the metabolic diversity, which may promote pathogenic infections or induce adverse metabolic effects for the host [142]. Several factors can influence the gut microbiome in patients with IF. An extensive small-bowel resection alters the intestinal environment, including the luminal pH and oxygen concentration, and the enterohepatic circulation of the bile acids [208]. The removal of the ICV predisposes the small intestine to overgrow bacteria, and removal of the ileum may lead to bile acid malabsorption. Bile acids have an antimicrobial activity and may lead to a relative abundance of *Firmicutes* at the expense of *Bacteroidetes* [177].

During the phase of intestinal adaptation, oral/enteral nutrition (EN) is initiated as soon as possible to stimulate the intestinal function. However, both the type and consequently the composition of oral nutrition or EN may also have an influence on the gut microbiota’s structure and function. In addition, feeding tubes may act as loci for bacterial attachment and biofilm formation. If no EN or oral nutrition is given, this has a substantial impact too. Ralls et al. [209] showed that EN deprivation in patients undergoing small-bowel resection (some receiving PN) led to the overabundance of *Proteobacteria*, which may be caused by a lack of dietary fermentable substrate (mainly fiber and resistance starch) in the gut lumen, necessary for the growth of certain dominant species. This “gut starvation” effect and the lack of interspecies competition offers the opportunity for subdominant species in the microbial community to increase over its dominant members. In patients with SBS and subsequent IF, antibiotics are often used to treat small-intestinal bacterial overgrowth or central line-associated bloodstream infections, which can influence the gut microbiome. Next to antibiotics, other medications frequently used in IF, such as proton pump inhibitors, can also alter the gut microbiome [177].

To date, there are no guidelines on the optimal timing for the transition from PN to EN, and there is not an ideal marker to use at present [193]. In the case of patients suffering from SBS and/or IF with a PN treatment, changes in the gut microbiome during gut adaptation may potentially be used as biomarkers to judge the optimal time of transition from PN to EN. Potent interventions to manipulate the gut microbiome include the use of pharmacological doses of SCFAs, prebiotics, probiotics, antibiotics and a fecal transplantation. SCFAs such as acetate, propionate and butyrate have the ability to promote cell proliferation and the differentiation of colonocytes, prevent the growth of opportunistic pathogens and are key regulators of the immune response [161,177]. Moreover, it might also be beneficial to use SCFAs as a trophic factor to stimulate and promote intestinal adaptation. Previous studies in animals showed that the supplementation of PN solutions with butyrate or mixed SCFAs may enhance the intestinal adaptation, an effect mediated by the upregulation of glucagon like peptide-2. However, the role of SCFAs in this process is not fully well established. Limited evidence suggests that synbiotics may increase the fecal levels of SCFAs as well as *Bifidobacteria*, total facultative anaerobic bacteria, *Enterobacteriaceae* and *Lactobacilli*; however, cases of bacteremia with prescribed probiotic bacteria in infants with SBS have also been reported. The efficacy of probiotics to promote an intestinal adaptation in patients with SBS is emerging, but evidence of their benefits remains limited; thus, the routine use of probiotics is currently not recommended in clinical practice [161,177]. Some studies have showed a reduction in pathogenic overgrowth and an improved growth and nutrition status in SBS patients treated with probiotics and/or synbiotics, although other researchers found no consistent positive or adverse effects of probiotics [194].

In summary, patients with SBS and/or IF have an altered gut microbiome and altered metabolic activity [161]. Despite differences in the primary pathology and underlying disease, the pattern of gut microbiota dysbiosis is very similar with profound shifts with an increase in *Proteobacteria*, especially *Enterobacteriaceae*, and a decrease in *Bacteroidetes* and often *Firmicutes.* Interestingly, specific changes in the gut microbiota’s composition can occur depending on the GI location which is affected [177]. Bacterial diversity is remarkably decreased, and there is a high abundance of *Lactobacillus*. Differences in microbiome characteristics have been found between patients receiving PN and those whose guts have adapted and have been weaned off PN. There is potential to use the gut microbiome as a biomarker to guide clinical practice during intestinal adaptation, as well as a modifiable therapeutic target.

## 4. Conclusions, Challenges and Future Directions

Evidence-based guidelines support PN as a complex form of intravenous therapy that has a live-saving potential in those patients for whom oral/enteral feeding is not possible. However, its long-term use also poses many challenges and serious complications in pediatric and critically ill patients, particularly in preterm infants, due to their gut immaturity and associated congenital or acquired gut disorders. The complications related to the use of central venous catheters, PN composition (in terms of sources and amounts of intravenous lipid emulsions), metabolic complications and growth retardation have also represented significant challenges for ICU pediatricians in optimizing long-term PN programs.

Recently, scientific and clinical interest has focused on evaluating the potential adverse effects of TPN on the composition and function of the gut microbiota as a cause or consequence of TPN-related comorbidities including inflammatory bowel disease, NEC, PNALD, gut mucosal atrophy, SBS and IF. In this review, we synthesized the available evidence from clinical trials that evaluated the relationships between gut microbiota dysbiosis and clinical outcomes in pediatric and preterm patients, and discussed the potential role of prebiotics, probiotics, synbiotics, paraprobiotics and postbiotics as promising strategies to reduce the aforementioned TPN-related comorbidities. The results obtained to date from human studies and animal models seem to suggest that long-term TPN causes profound gut microbiota dysbiosis, which is mainly characterized by a decreased α-diversity, the lower abundance of commensal bacteria as well as the increased prevalence of potentially pathogenic Gram-negative bacteria. However, a common pattern of gut microbiota dysbiosis has not been yet identified, so the mechanistic role of gut microbiota in disease incidence and progression remains unclear. Nevertheless, these findings open the possibility of using promising gut microbiota-based therapies for TPN-related diseases. Unfortunately, a broad consensus for the majority of indications, specific strains, dosages and treatment regimens is lacking. In light of these findings, the recent ESPGHAN guidelines make only weak recommendations for the use of this treatment in pediatric patients, thus supporting the need for more well-designed, large, strain-specific and dedicated dose–response studies.

Having these considerations in mind, many research gaps and questions need to be solved in the topic of TPN-related diseases and gut microbiota in the pediatric population. In this sense, future researchers will need to: (a) identify the patterns of gut microbiota dysbiosis and GI location-specific changes in TPN-related diseases, and determine whether changes in the composition of the gut microbiota are a cause or a consequence of these diseases; (b) better understand the role of pre- or probiotics and synbiotics as well as other promising gut microbiota-based treatments such as postbiotics and paraprobiotics, as therapeutic tools and their mechanisms of action on the gut microbiota; (c) include metabolite and gene expression profiling, in addition to microbiome sequencing; and (d) expand their research to the full microbial community, not only based on changes in the gut microbiota’s composition. In order to achieve these aims, specific methodological challenges must be overcome, mainly related to: (1) the standardization and harmonization of protocols for microbiome analysis; (2) moving from animal models to human studies; and (3) including an appropriate sample size and power calculations for microbiome studies, which must be used in well-designed, large randomized controlled trials. Undoubtedly, the routinary use of new molecular techniques and multiomic approaches acquires a vital importance in facing these challenges. To date, its application has enabled us to evaluate in depth both the structure and functional activity of the gut microbiota in animal models and human studies. Nevertheless, these approaches must be also considered as promising tools to better understand how changes in the composition and activity of the gut microbiota have an influence on the health outcomes [210]. Finally, the resolution of these challenges and questions will allow us to design individualized strategies which are focused on the gut microbiota’s composition and function for the prevention of TPN-related gastrointestinal diseases in the pediatric population, improving their clinical outcomes when long-term PN is required. In this regard, for instance, there are still many challenges regarding the safety and effectiveness of a fecal microbiome transplant in order to restore the gut microbiota in humans, although promising short-term clinical outcomes have been obtained in animal models of SBS fed with PN [211]. Interestingly, this new knowledge must be considered in updated guidelines in order to strongly recommend the use of prebiotics, probiotics, synbiotics, postbiotics and paraprobiotics as an adjunctive therapy in the prevention and treatment of PN-related diseases.

## Data Availability

Not applicable.
